# Discriminative power of diverse nonlinear EEG dynamics across consciousness states during auditory stimulation in disorders of consciousness

**DOI:** 10.3389/fnhum.2025.1640520

**Published:** 2025-09-12

**Authors:** Yaxiu Tang, Qu Sheng, Xinchun Wu, Fanshuo Zeng

**Affiliations:** Department of Rehabilitation, The Second Qilu Hospital of Shandong University, Jinan, China

**Keywords:** disorder of consciousness, electroencephalogram, auditory stimuli, Lyapunov exponent, approximate entropy, Lempel–Ziv complexity, correlation dimension

## Abstract

**Objective:**

This study addressed the challenge of assessing consciousness in patients with disorders of consciousness (DOC) using nonlinear dynamic parameters applied to electroencephalogram (EEG) characteristics stimulated by music.

**Method:**

EEG signals from 57 patients with DOC at the Rehabilitation Medical Center of the Second Hospital of Shandong University were analyzed using the Lyapunov Exponent (LE), Approximate Entropy (ApEn), Lempel–Ziv Complexity (LZC), and Correlation Dimension (D2). These parameters were then correlated with the total CRS-R score to evaluate their effectiveness. The results indicated that preferred music significantly improved cortical excitability in minimally conscious state (MCS) patients, as evidenced by the ApEn, LZC, and D2 algorithms, but had no significant effect on vegetative state (VS)/unresponsive wakefulness syndrome (UWS) patients.

**Results:**

LE, however, showed significant responsiveness in the frontal and middle temporal lobes of patients with VS/UWS. Correlation analysis revealed significant associations between changes in ApEn, LZC, and D2 due to musical stimulation and the total CRS-R score, whereas the LE showed no such correlation.

**Conclusion:**

ApEn, LZC, and D2 are valuable for assessing and distinguishing conscious states in patients with DOC, with LE providing supplementary information for patients with VS/UWS. Nonlinear EEG parameters offer promising reference values for effective clinical interventions in DOC.

## 1 Introduction

After severe acquired brain injury, some patients may exhibit a persistent consciousness (pDOC) that lasts for more than 28 days (Wang et al., 2020). Depending on the level of preserved consciousness, these patients are typically classified into two main categories: vegetative state (VS)/unresponsive wakefulness syndrome (UWS), characterized by spontaneous eye-opening without any conscious behavior, and minimally conscious state (MCS), characterized by inconsistent but reproducible conscious behavior (Ling et al., 2023).

In recent years, clinical research has focused on identifying effective methods to promote awakening in patients with disorders of consciousness (DOC). One such method is musical stimulation, which has been widely used to prevent sensory deprivation in patients by potentially affecting neural networks and accelerating neuronal plasticity (Herdener et al., 2010). In addition, musical stimulation has been shown to activate a wide range of cortical responses, supporting concepts such as the “Mozart effect” and the “mood and arousal hypothesis” (Luauté et al., 2018).

EEG (Electroencephalogram) is a non-invasive brain function testing technique known for its speed, convenience, and ease of bedside assessment. It offers high temporal and acceptable spatial resolution (Lai et al., 2020), making it an ideal choice for this study to objectively assess patients with DOC in the presence of musical stimuli. Traditionally, EEG signal features have been extracted using linear methods in the frequency domain (e.g., fast Fourier transform or wavelet transform) and parametric techniques (e.g., autoregressive modeling). However, these linear methods are limited in their ability to detect potentially nonlinear features (Pritchard and Duke, 1995). EEG signals exhibit significant random fluctuations in amplitude over time and are characterized by nonlinear and dynamic features (Akdemir Akar et al., 2015; Liu et al., 2021). Therefore, it is important to use nonlinear dynamics to extract EEG signal features, as these are fundamentally independent of EEG spectral analysis parameters and are complementary to “classical” EEG linear analysis (Eagleman and Drover, 2018; Altıntop et al., 2022). Nonlinear analyses are believed to reveal the complexity of EEG signals through various measures, such as the Lyapunov Exponent (LE), Approximate Entropy (ApEn), Lempel–Ziv Complexity (LZC), and Correlation Dimension (D2). Each of these algorithms captures different aspects of the signal complexity. We hypothesized that using these four algorithms to assess the effects of musical stimuli on the cerebral cortex in patients with DOC would yield different results. Therefore, this study aimed to identify the nonlinear dynamic algorithms that most accurately assess the state of consciousness in patients with DOCs under quiet and external stimulation conditions.

## 2 Materials and methods

### 2.1 Study design

This prospective cross-sectional study was conducted from October 17, 2023, to May 20, 2024. A total of 57 patients with DOC and 22 healthy participants from the Department of Rehabilitation Medicine of the Second Hospital of Shandong University participated in this study. The study was approved by the Ethics Committee of the Second Hospital of Shandong University (KYLL-2023-414) and the registration code was ChiCTR2300079310. Informed consent was obtained from each patients’ family.

The VS/UWS state is defined as the presence of an awake state without responsiveness to external stimuli. Patients with pDOC and MCS exhibit fluctuating responses to external stimuli. Patients with MCS are further subdivided into MCS + and MCS- based on their ability to process language (Ling et al., 2023). Patients with MCS exhibit intermittent responses to external stimuli, specifically primary or reflexive behaviors such as body flinching after a painful stimulus or eye tracking in response to mirrors. Conversely, MCS + patients could use objects normally and communicate effectively and reliably with their surroundings. The inclusion criteria were: 1. The diagnostic criteria for DOC were met: 2. Aged 18–80 years; 3. no previous history of brain injury, and 4. Stable vital signs. Exclusion criteria were as follows: 1. Hearing impairment; 2. continuous aggravation, active cerebral hemorrhage, or intracranial hypertension, and 3. Atresia syndrome, severe cerebral atrophy, or hydrocephalus; 4. History of epilepsy, 5. severe spasticity resulting in EMG artifacts, and 6. cranial decompressive resection or cranial defects affecting regional EEG amplitude, and 7. Neurodevelopmental disorders.

### 2.2 General parameters

Patient clinical data, including age, sex, duration of injury, and type of injury (traumatic brain injury, stroke, or ischemic–hypoxic encephalopathy), were collected. The behavioral assessment of the patients was performed using the Coma Recovery Scale-Revised (CRS-R). The CRS-R is considered the most valid and sensitive method for identifying individuals with very low consciousness (Cortese et al., 2015). This scale consists of six subscales: auditory, visual, motor, verbal, communicative, and arousal (Wang et al., 2020). CRS-R assessments are usually performed between 10 a.m. and 12 p.m. (Cortese et al., 2015) and separated from the administration of CNS medications (e.g., muscle relaxants and sedatives) by an interval of at least 10 h. An experienced rehabilitative physician conducted the EEG assessments on the same day. All the CRS-R evaluations were conducted by an experienced physician who was blinded to the EEG results throughout the assessment process. To enhance reliability, each patient underwent three consecutive CRS-R assessments (on the EEG recording day and subsequent 2 days), with the average score used for statistical analysis. The study employed separate specialized teams for different components: EEG acquisition by trained neurophysiological technicians. EEG analysis by dedicated signal processing researchers. Behavioral assessments by clinical rehabilitation staff.

### 2.3 EEG examination

For this study, EEG signal acquisition (ZN16E, Chengdu, China) was performed in the unipolar mode, which records the voltage difference between the measurement and reference electrodes. Multiple electrodes were used to record neuroelectric activity across different brain regions. A wireless digital EEG amplifier and a powerless chamber were used to minimize electromagnetic interference from the surrounding environment. EEG recordings were obtained while the patient was awake and lying comfortably in a quiet environment. All electrodes referenced earlobe electrodes to record EEG from 19 scalp sites (channels Fp1, Fp2, F3, F4, C3, C4, P3, P4, O1, O2, F7, F8, T3, T4, T5, T6, Fz, Cz, and Pz), according to the International 10–20 systems. The signals were digitized at a sample rate of 500 Hz, bandwidth of 0.3–100 Hz, and 12-bit AD conversion resolution.

Initially, EEG signals were recorded in a quiet state for 5 min. Subsequently, EEG signals were recorded for another 5 min while patients listened to their preferred music, which was obtained through interviews with the patients’ family members. To prevent emotional bias, the selected music was upbeaten and optimistic. All music was played using binaural noise-canceling headphones at a volume of 60–70 dB.

To ensure that the patient remained awake during the test, an experienced EEG evaluator monitored EEG traces online for signs of sleepiness and sleep onset. Vigilance states were classified according to standardized electrophysiological criteria. Wakefulness was defined as sustained posterior dominant rhythm in either the alpha (8–12 Hz) or low-beta (12–18 Hz) frequency ranges, with absence of sleep-specific patterns including sleep spindles and K-complexes. If signs of behavioral sleepiness and/or EEG sleepiness were detected, the assessment was paused, and the patient was prodded awake according to the CRS-R standard wakefulness pattern. EEG signals from the corresponding segments were then recaptured. Participants were asked to keep their eyes closed throughout the procedure to minimize eye movement artifacts. To minimize electromyographic artifacts, participants with obvious spasticity were excluded, and EEG signals mixed with electromyographic artifacts were visually excluded by experienced EEG operators. Because ApEn and LZC are highly sensitive to high-frequency components in EEG signals (Kargarnovin et al., 2023), data showing a significant increase in nonlinear indexes due to invisible EMG (in the range of 50–150 Hz) were excluded. Ultimately, 65,536 consecutive intervals were selected for further analysis. For analysis, a 50 Hz trap filter was used to remove electrical noise, a 70 Hz high-frequency filter to reduce myoelectric interference, and a 0.53 Hz low-frequency filter to attenuate artifacts.

### 2.4 Nonlinear parameters

#### 2.4.1 Lyapunov exponent

The LE is used to estimate the divergence (positive exponent) or convergence (negative exponent) of two neighboring trajectories in phase space. A positive Lyapunov Exponent indicates that the studied system is chaotic. Unlike the static D2 measure, LE provides a measure of relative dynamism, making it more suitable for assessing the brain’s ability to process information flexibly and to develop and evolve different information processing states from a similar initial state. The first dynamic system is formulated as follows:


$$\dot{x}=f\;(x),$$

Where X is the state vector in N dimensions.

We selected two phase points in the phase space, plotted their trajectories [ x1(t) and x2(t) ], and tracked the changes in the distances d between corresponding points of these trajectories during the evolution of the system. LE was calculated as follows:


$$d(t)=|\vec{\varepsilon }(t)|=|x_{2}(t)-x_{1}(t)|$$

$$d(t)\approx d(0)e^{\mathrm{kt}}$$

$$k=\begin{array}{c} \lim\\ \underset{t\to \infty }{d(0)\to 0} \end{array}\;\frac{\text{In}[d(t)/d(0)]}{t}$$

The h value (sum of positive exponents) is called the Kolmogorov–Sinai entropy or ks-entropy. Using ks-entropy, it is possible to determine whether the model under study is chaotic or regular. For chaotic systems, a ks-entropy value greater than 0 indicates chaos, while a value of 0 or less indicates a regular system.

#### 2.4.2 Approximate entropy

ApEn, proposed by Pincus (2000), quantifies the unpredictability or randomness of a signal. It originates from nonlinear dynamics and is closely related to the LE, reflecting the rate of loss of information about the dynamics of a nervous system over time. ApEn is robust to low-frequency noise (Pincus, 2000; Richman and Moorman, 2000; Pincus, 2006) and suitable for time series that are relatively short (>100 data points) and mixed with noise. The calculation formula is as follows:


$$\begin{array}{l} |X_{i+k}-X_{i+k}|<r,0\leq k\leq m\\ \text{ApEn}\;(m,r,N)=\text{In}\frac{C_{m}(r)}{C_{m+1}(r)}\\ \mathrm{Ci},m\;(r)=\frac{n_{i},m\;(r)}{N-m+1} \end{array}$$

The absolute value of ApEn is affected by three parameters: the elapsed time (N), the number of previous values used to predict subsequent values (m), and the filtering level (r). For this study, N was fixed at 4096 to improve the accuracy of the analysis. The noise filter defines the tolerance r, which is used to discriminate between “close” and “not close” subvectors of length N. The Filter level r was used to measure the amount of noise in the filtered data. Typically, r is selected based on the standard deviation (SD) of the signal. Referring to Ferenets et al., *r* = 0.2 SD and *m* = 2 were set for this study.

#### 2.4.3 Lempel–Ziv complexity

The LZC algorithm comprises two main steps: coarse-graining and complexity processing (Ibáñez-Molina et al., 2015). First, the LZC of the EEG signal segment (N: segmentation length) is computed by converting the EEG data into a binary string (s(n)) (Maschke et al., 2022):


$$s(n)=\{\begin{array}{c} 1,if\;x\left(n\right)>T\\ 0,if\;x\left(n\right)>T \end{array}$$

Second, after the binary classification process, the binary string is analyzed from the beginning to produce a new sequence of symbols c(n). LZC computes the number of different patterns in the binary string and the complexity b(n) for length segments using an upper bound on the complexity c(n), which converges to a constant value for almost all c(n) values after computation:


$$\lim_{n\to \infty }c(n)=b(n)=\frac{n}{\log_{2}n}\;\;\mathrm{Lz}=\frac{c(n)}{b(n)}$$

LZC typically ranges from 0 to 1, representing a nonlinear dynamic measure that indicates the rate at which new patterns emerge in a time series. A larger LZC implies a greater likelihood of new sequence patterns emerging, signifying more complex dynamic behavior. When n is large, LZC becomes independent of the number of samples. To ensure accuracy, n was fixed at 4096 for this study.

#### 2.4.4 Correlation dimension

The D2 determines and assesses the complexity of the system’s dynamics by using individual time series. This is achieved by constructing a pseudo-attractor in phase space using the attractor measurement properties of the system (Carlino et al., 2012). The pseudo-attractor is then used to compute D2 without reconstructing the dynamic system from which the time series is generated. D2 characterizes the dynamics of the EEG signal, providing information about the chaotic degrees of freedom and expressing the complexity of the system. The dimension of association (D2) is calculated as follows (Molnár et al., 1997):


$$\begin{array}{l} D_{2}=\lim_{r\to 0}\frac{\log C(r)}{\log(r)}\\ C(r)=\frac{1}{N_{\mathrm{re}}}\sum_{i=1}^{N_{\mathrm{ref}}}\frac{1}{N-i}\sum_{j>i}^{N}\theta [r-|V(j)-V(i)|] \end{array}$$

### 2.5 Statistical analysis

Data analysis was performed using IBM SPSS for Windows (version 26.0; IBM Corp, Armonk, NY, United States). The normality of the measurement data was assessed using the Shapiro–Wilk test. Continuous variables that followed a normal distribution were expressed as mean±standard deviation and compared between groups using the two independent samples t-test. Discrete variables that did not follow a normal distribution were expressed as quartiles (IQR) and compared using the Mann–Whitney U test. Categorical variables were described as percentages (%) and compared using the chi-square test.

Within-group comparisons using paired t-tests to assess music-induced cortical activity in nonlinear parameters (LE, ApEn, LZC, D2) between resting and post-music stimulation states across VS/UWS, MCS, and healthy subject groups. Between-group analyses employing one-way ANOVA with Bonferroni correction to compare these groups, respectively, in both resting and music conditions. Furthermore, MCS subgroup analyses featured: one-way ANOVA for between-subgroup differences in both resting and music conditions, respectively. The relationships were assessed using Pearson’s correlation analysis. Correlation coefficients were interpreted according to established thresholds: strong (0.6 ≤ *R* < 0.8), moderate (0.4 ≤ *R* < 0.6), and weak (0.2 ≤ *R* < 0.4) associations between the mean differences and CRS-R total scores. A *p*-value of less than 0.05 was considered statistically significant for all analyses.

## 3 Results

### 3.1 Clinical baseline information

This study included a total of 59 patients with DOC, comprising 28 patients with VS/UWS and 31 patients with MCS. Due to severe motor artifacts, the EEG signals of two patients with DOC were excluded, resulting in a final DOC sample size of 57 patients (28 VS/UWS and 29 MCS). In addition, 22 healthy participants were included, bringing the total sample size to 79. The healthy participants consisted of 15 males and 7 females with a median age of 60 years (IQR: 51.00, 70.00). The VS/UWS group included 21 males and 7 females with a median age of 61.5 years (IQR: 45.00, 71.75) and a time to injury of 72 years (IQR: 55.25, 104.50). The MCS group included 23 males and 6 females with a median age of 60 years (IQR: 47.50, 73.00) and time to injury of 90 years (IQR: 45.00, 180.50). In addition, the median CRS-R total score was 4.00 (IQR: 2.00, 6.00) in the VS/UWS group and 9.00 (7.50, 10.00) in the MCS group, indicating significantly higher scores in the MCS group compared to the VS/UWS group. There were no statistically significant differences between the VS/UWS and MCS groups in terms of age, sex, duration of injury, or type of injury (*p* > 0.05), as shown in Table 1.

**Table 1:** Demographic characteristics of DOC patients.

Feature	VS/UWS group (*n* = 28)	MCS group (*n* = 29)	Healthy control group (*n* = 22)
Age (y)	61.50 (45.00, 71.75)	60.00 (47.50, 73.00)	60.0 (51.00, 70.00)
Days post-injury	72.00 (55.25, 104.50)	90.00 (45.00, 180.50)	
Sex			
Male	21	23	15
Female	7	6	7
Etiology			
Traumatic brain injury	4	8	
Stroke	22	20	
Hypoxic brain injury	2	1	
CRS-R scores	4.00 (2.00, 6.00)	9.00 (7.50, 10.00)	

Coma Recovery Scale-Revised (CRS-R), VS/UWS, Vegetative state/unresponsive wakefulness syndrome; MCS, Minimally conscious state.

### 3.2 Intergroup differences in nonlinear cortical responses to musical stimulation

**Figure 1:**
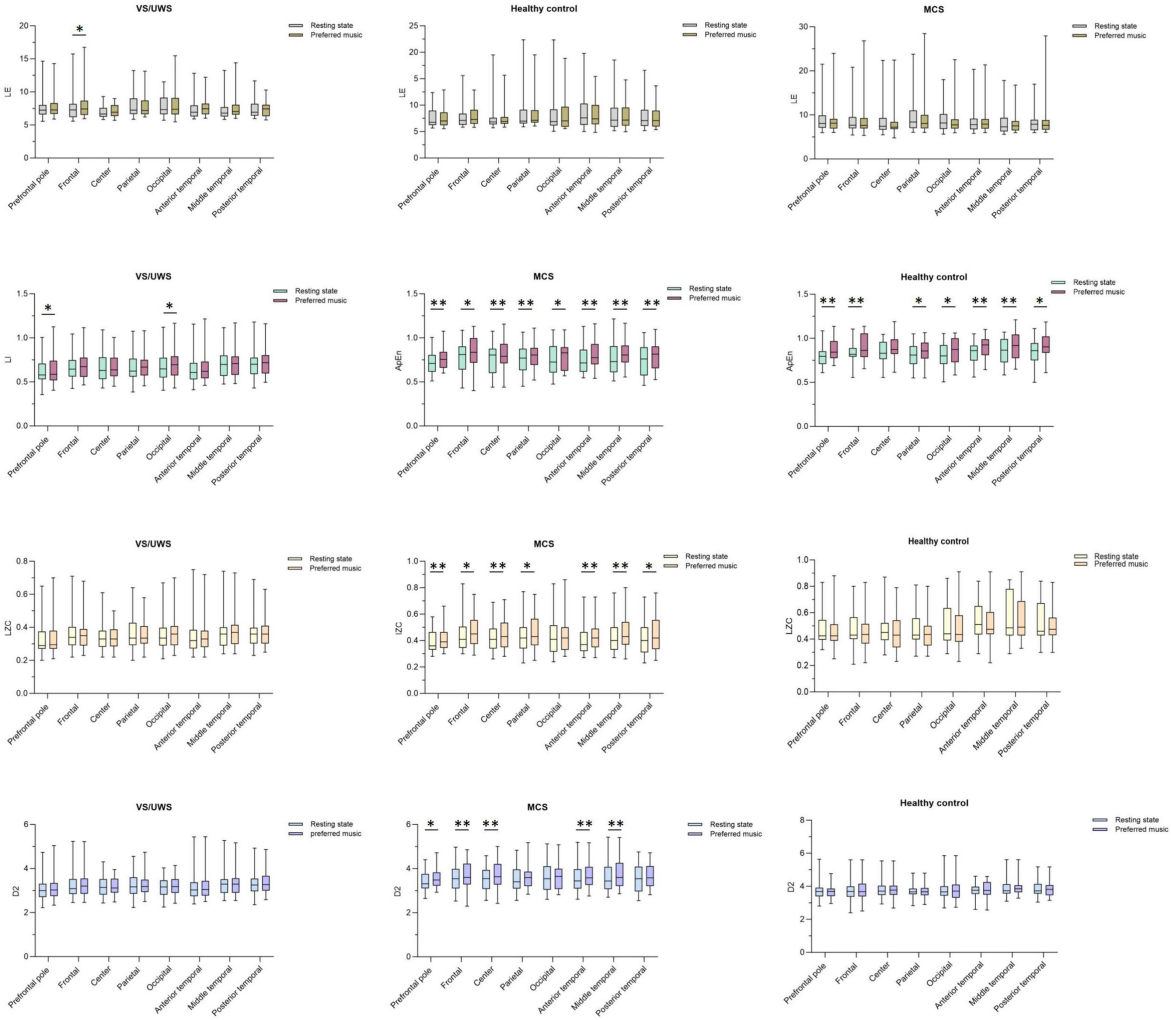
Four Nonlinear Dynamics in Three Population Groups. Statistical comparison of nonlinear measures (LE, ApEn, LZC, D2) between resting state and music stimulation conditions in healthy controls and patients with VS/UWS or MCS was performed using paired-samples t-tests: ^*^p < 0.05, ^**^p < 0.01. VS/UWS, Vegetative state/unresponsive wakefulness syndrome; MCS, Minimally conscious state; LE, Lyapunov Exponent; ApEn, Approximate Entropy; LZC, Lempel-Ziv Complexity; D2, Correlation Dimension D2.

The application of various nonlinear parameters provided detailed insights into the effects of musical stimulation on cortical excitability across different groups. LE nonlinear analysis showed that the frontal lobes of the VS/UWS group exhibited a significant increase in cortical excitability following musical stimulation. In contrast, no significant response to musical stimulation was observed in any brain regions of the MCS group or the healthy control group. ApEn nonlinear analysis revealed that musical stimulation increased cortical excitability in prefrontal pole and occipital regions of the VS/UWS group. Conversely, musical stimulation significantly increased the excitability of the entire cerebral cortex in the MCS group, including the prefrontal pole and the frontal, central, parietal, occipital, anterior temporal, middle temporal, and posterior temporal lobes. In the healthy group, musical stimulation significantly increased excitability in the prefrontal pole and the frontal, parietal, occipital, anterior temporal, middle temporal, and posterior temporal lobes. The results of the LZC nonlinear analysis revealed that the VS/UWS and healthy control groups showed no significant responsiveness to musical stimulation. However, the MCS group demonstrated significant responsiveness in the prefrontal pole, frontal, central, parietal, and middle temporal lobes. D2 nonlinear analysis showed no significant activation in the VS/UWS and healthy control groups. In the MCS group, musical stimulation activated the prefrontal pole, frontal, central, anterior temporal, and middle temporal lobes (Table 2; Figure 1).

**Table 2:** Effects of preferred music on EEG nonlinear parameters.

Nonlinear parameters	VS/UWS group (*n* = 8)			MCS group (*n* = 29)			Healthy subject group (*n* = 22)		
	Resting state	Preferred music	Paired Cohen’s d	Resting state	Preferred music	Paired Cohen’s d	Resting state	Preferred music	Paired Cohen’s d
LE									
Prefrontal pole	7.70 ± 1.93	7.66 ± 1.67	−0.06	8.84 ± 3.06	8.72 ± 3.35	−0.12	7.64 ± 2.06	7.72 ± 2.14	0.11
Frontal	**7.69 ± 2.05** ^**h**^	8.60 ± 3.19	0.36	8.91 ± 3.55	8.87 ± 4.12	−0.03	7.85 ± 2.37	7.88 ± 2.00	0.03
Center	7.02 ± 1.02	7.09 ± 0.89	0.11	8.50 ± 3.75	8.29 ± 3.69	−0.29	7.78 ± 2.98	7.69 ± 2.27	−0.09
Parietal	8.04 ± 2.02	7.90 ± 1.69	−0.24	9.62 ± 4.04	9.30 ± 4.26	−0.18	8.54 ± 3.77	8.55 ± 3.30	0.01
Occipital	7.74 ± 1.56	7.87 ± 1.95	0.13	8.72 ± 2.63	8.57 ± 3.09	−0.11	8.53 ± 4.14	8.44 ± 3.60	−0.08
Anterior temporal	7.42 ± 1.50	7.61 ± 1.33	0.35	8.59 ± 2.98	8.53 ± 2.91	−0.07	8.42 ± 3.23	8.32 ± 2.62	−0.07
Middle temporal	7.21 ± 1.44	7.45 ± 1.61	0.52	8.05 ± 2.49	7.96 ± 2.21	−0.14	8.10 ± 3.00	8.04 ± 2.49	−0.06
Posterior temporal	7.38 ± 1.36	7.45 ± 1.19	0.10	8.33 ± 2.74	8.61 ± 4.17	0.12	7.85 ± 2.62	7.73 ± 2.31	−0.11
ApEn	1	4		2	5		3	6	
Prefrontal pole	**0.61 ± 0.13** ^**aah**^	**0.63 ± 0.15** ^**dd**^	0.43	**0.72 ± 0.72** ^**ii**^	**0.76 ± 0.12** ^**f**^	0.83	**0.81 ± 0.12** ^**bbgg**^	**0.87 ± 0.13** ^**ee**^	0.81
Frontal	**0.67 ± 0.15** ^**a**^	**0.69 ± 0.15** ^**dd**^	0.28	**0.78 ± 0.17** ^**i**^	0.83 ± 0.17	0.47	**0.82 ± 0.13** ^**bbgg**^	**0.88 ± 0.17** ^**ee**^	0.62
Center	0.67 ± 0.15	**0.66 ± 0.14** ^**dd**^	−0.02	**0.76 ± 0.17** ^**ii**^	0.83 ± 0.17	0.67	**0.82 ± 0.14** ^**bb**^	**0.87 ± 0.17** ^**ee**^	0.38
Parietal	0.68 ± 0.16	**0.68 ± 0.15** ^**d**^	0.08	**0.75 ± 0.17** ^**ii**^	0.80 ± 0.15	0.60	**0.79 ± 0.13** ^**bbg**^	**0.83 ± 0.15** ^**ee**^	0.46
Occipital	**0.68 ± 0.15** ^**h**^	0.70 ± 0.16	0.42	**0.77 ± 0.17** ^**i**^	0.80 ± 0.16	0.51	**0.80 ± 0.14** ^**bg**^	**0.84 ± 0.17** ^**ee**^	0.47
Anterior temporal	**0.64 ± 0.16** ^**a**^	**0.66 ± 0.15** ^**dd**^	0.27	**0.77 ± 0.18** ^**cii**^	0.82 ± 0.16	0.77	**0.85 ± 0.13** ^**bbgg**^	**0.90 ± 0.12** ^**ee**^	0.71
Middle temporal	0.70 ± 0.15	**0.70 ± 0.15** ^**d**^	0.09	**0.77 ± 0.20** ^**ii**^	0.83 ± 0.16	0.55	**0.86 ± 0.15** ^**bbgg**^	**0.92 ± 0.16** ^**ee**^	0.66
Posterior temporal	0.70 ± 0.16	0.72 ± 0.15	0.23	**0.75 ± 0.18** ^**ii**^	**0.80 ± 0.15** ^**f**^	0.60	**0.85 ± 0.15** ^**bbg**^	**0.90 ± 0.16** ^**ee**^	0.52
LZC									
Prefrontal pole	**0.32 ± 0.09** ^**a**^	**0.32 ± 0.10** ^**d**^	0.29	**0.39 ± 0.82** ^**cii**^	0.42 ± 0.09	0.70	**0.48 ± 0.15** ^**bb**^	**0.47 ± 0.15** ^**e**^	−0.13
Frontal	**0.35 ± 0.10** ^**a**^	**0.35 ± 0.09** ^**d**^	−0.04	**0.45 ± 0.15** ^**i**^	0.48 ± 0.12	0.39	**0.47 ± 0.16** ^**bb**^	**0.45 ± 0.14** ^**e**^	−0.22
Center	0.34 ± 0.80	**0.34 ± 0.07** ^**d**^	−0.09	**0.42 ± 0.11** ^**ii**^	0.45 ± 0.12	0.72	**0.48 ± 0.16** ^**bb**^	**0.44 ± 0.14** ^**e**^	−0.38
Parietal	0.36 ± 0.10	**0.36 ± 0.09** ^**d**^	−0.08	**0.43 ± 0.13** ^**i**^	0.45 ± 0.13	0.43	**0.48 ± 0.14** ^**bb**^	**0.46 ± 0.15** ^**e**^	−0.24
Occipital	0.36 ± 0.10	0.36 ± 0.10	0.26	0.43 ± 0.14	0.44 ± 0.14	0.25	**0.52 ± 0.19** ^**bb**^	**0.48 ± 0.18** ^**e**^	−0.32
Anterior temporal	0.34 ± 0.10	**0.34 ± 0.10** ^**d**^	0.13	**0.41 ± 0.13** ^**ccii**^	0.44 ± 0.12	0.71	**0.53 ± 0.17** ^**bb**^	**0.52 ± 0.17** ^**e**^	−0.07
Middle temporal	0.36 ± 0.10	**0.36 ± 0.10** ^**d**^	0.13	**0.42 ± 0.13** ^**ccii**^	**0.45 ± 0.13** ^**f**^	0.83	**0.56 ± 0.18** ^**bb**^	**0.55 ± 0.17** ^**e**^	−0.11
Posterior temporal	0.36 ± 0.10	0.37 ± 0.09	0.17	**0.42 ± 0.13** ^**cc**^	0.43 ± 0.12	0.31	**0.53 ± 0.15** ^**bb**^	**0.50 ± 0.15** ^**e**^	−0.29
D2									
Prefrontal pole	**3.03 ± 0.47** ^**a**^	**3.08 ± 0.52** ^**dd**^	0.27	**3.44 ± 0.46** ^**i**^	3.55 ± 0.45	0.49	**3.77 ± 0.61** ^**bb**^	**3.74 ± 0.40** ^**ee**^	0.09
Frontal	3.21 ± 0.57	**3.27 ± 0.57** ^**d**^	0.33	**3.60 ± 0.61** ^**ii**^	3.74 ± 0.62	0.55	**3.75 ± 0.75** ^**b**^	**3.82 ± 0.75** ^**e**^	0.36
Center	**3.18 ± 0.42** ^**a**^	**3.19 ± 0.39** ^**dd**^	0.02	**3.54 ± 0.56** ^**ii**^	3.69 ± 0.59	0.76	**3.81 ± 0.62** ^**bb**^	**3.84 ± 0.61** ^**ee**^	0.12
Parietal	3.24 ± 0.51	**3.26 ± 0.47** ^**d**^	0.08	3.55 ± 0.56	3.64 ± 0.58	0.33	**3.68 ± 0.47** ^**b**^	**3.68 ± 0.48** ^**e**^	0.01
Occipital	**3.17 ± 0.44** ^**a**^	**3.21 ± 0.43** ^**d**^	0.21	3.62 ± 0.66	3.67 ± 0.63	0.19	**3.79 ± 0.73** ^**bb**^	**3.80 ± 0.76** ^**ee**^	0.03
Anterior temporal	**3.13 ± 0.58** ^**a**^	**3.16 ± 0.56** ^**dd**^	0.18	**3.56 ± 0.65** ^**ii**^	3.70 ± 0.58	0.54	**3.76 ± 0.49** ^**bb**^	**3.80 ± 0.51** ^**ee**^	0.21
Middle temporal	3.30 ± 0.56	**3.32 ± 0.55** ^**d**^	0.06	**3.62 ± 0.71** ^**ii**^	3.79 ± 0.69	0.63	**3.91 ± 0.63** ^**bb**^	**3.94 ± 0.58** ^**ee**^	0.17
Posterior temporal	3.30 ± 0.50	3.34 ± 0.50	0.23	3.59 ± 0.63	3.64 ± 0.57	0.15	**3.87 ± 0.56** ^**bb**^	**3.86 ± 0.53** ^**ee**^	0.06

VS/UWS, Vegetative state/unresponsive wakefulness syndrome; MCS, Minimally conscious state; LE, Lyapunov Exponent, ApEn, Approximate Entropy, LZC, Lempel–Ziv Complexity; D2, Correlation Dimension D2. The ratio of the VS/UWS group to the MCS group in resting state, Bold ^a^*p* < 0.05. The ratio of VS/UWS group to healthy participants in resting state, Bold ^b^*p* < 0.05. The ratio of the MCS group to the healthy participant group in resting state, Bold ^c^*p* < 0.05. The ratio of the VS/UWS to MCS group in preferred music, Bold ^d^*p* < 0.05. The ratio of the VS/UWS group to healthy participants in preferred music, Bold ^e^*p* < 0.05. The ratio of the MCS group to the healthy participant group in preferred music, Bold ^f^*p* < 0.05. The ratio of the VS/UWS group in resting state and preferred music, Bold ^h^*p* < 0.05. The ratio of the MCS group between resting state and preferred music, Bold ^i^*p* < 0.05. The ratio of the healthy participant group between resting state and preferred music, Bold ^g^*p* < 0.05.

### 3.3 Comparison of differences in nonlinear parameters among the three groups both in the resting state and music stimulation

Our nonlinear EEG analysis revealed distinct consciousness-state-dependent patterns across metrics. LE analysis showed no significant group differences in neural excitability during either resting state or musical stimulation (all *p* > 0.05). However, resting-state analyses demonstrated hierarchical complexity reductions: All three metrics (ApEn/LZC/D2) revealed significantly reduced complexity in VS/UWS versus controls globally (all *p* < 0.05), with region-specific impairments versus MCS: ApEn/D2 in prefrontal pole/occipital/anterior temporal areas (all *p* < 0.05), and LZC in most regions except prefrontal/frontal. MCS patients showed intermediate ApEn/D2 with anterior temporal/prefrontal pole and temporal deficits versus controls (*p* < 0.05), while maintaining near-normal LZC values. During musical stimulation, VS/UWS displayed: (1) global ApEn/LZC/D2 reductions, (2) both ApEn and D2 exhibited widespread reductions (prefrontal pole/frontal/central/parietal/anterior/middle temporal vs. MCS), while LZC showed global impairment (all regions *p* < 0.05 except posterior temporal vs. MCS). In contrast, MCS patients maintained preserved complexity metrics overall, with only localized reductions in prefrontal/posterior temporal ApEn and middle temporal LZC (all *p* < 0.05), while D2 values showed no significant differences versus healthy controls.

### 3.4 Comparison of nonlinear parameters between MCS- and MCS + in resting state and during musical stimulation

In both the resting state and during musical stimulation, MCS + patients exhibited higher cortical excitability compared to MCS- patients. The ApEn and D2 nonlinear analysis methods indicated that in the quiet state, excitability in the occipital cortex was significantly higher in MCS + patients than in MCS- patients (*p* < 0.05). In contrast, the LE nonlinear analysis revealed that in the quiet state, excitability in the central, parietal, and occipital cortices was significantly higher in MCS **+** patients compared to MCS- patients. Additionally, the MCS-parietal and occipital cortex excitability was significantly higher in MCS- patients (*p* < 0.05). Notably, the LZC nonlinear analysis method more effectively revealed differences in cortical excitability between MCS- and MCS + patients. It showed that excitability in the central, parietal, occipital, and posterior temporal cortex was significantly higher in MCS + patients compared to MCS- patients in both quiet and preferred music states (*p* < 0.05; Table 3).

**Table 3:** Differences on different nonlinear dynamics between MCS− and MCS+ after preferred music.

Nonlinear parameters	Resting state		Preferred music	
	MCS− (*n* = 17)	MCS+ (*n* = 12)	MCS− (*n* = 17)	MCS+ (*n* = 12)
LE				
Prefrontal pole	7.96 ± 1.52	10.09 ± 4.19	7.74 ± 1.20	10.10 ± 4.78
Frontal	8.11 ± 1.89	10.03 ± 4.95	7.89 ± 1.41	10.27 ± 6.05
Center	**7.36 ± 1.13** ^**a**^	10.10 ± 5.39	7.29 ± 1.05	9.71 ± 5.42
Parietal	**8.10 ± 2.04** ^**a**^	11.76 ± 5.20	**7.93 ± 1.66** ^**b**^	11.23 ± 5.93
Occipital	**7.69 ± 1.80** ^**a**^	10.17 ± 2.99	**7.53 ± 1.41** ^**b**^	10.05 ± 4.16
Anterior temporal	7.98 ± 1.51	9.45 ± 4.23	7.88 ± 1.35	9.46 ± 4.16
Middle temporal	7.57 ± 1.60	8.74 ± 3.36	7.56 ± 1.33	8.53 ± 3.04
Posterior temporal	7.59 ± 1.85	9.38 ± 3.49	7.42 ± 1.31	10.30 ± 6.03
ApEn				
Prefrontal pole	0.70 ± 0.10	0.76 ± 0.15	0.74 ± 0.09	0.79 ± 0.14
Frontal	0.77 ± 0.14	0.80 ± 0.21	0.82 ± 0.15	0.84 ± 0.20
Center	0.74 ± 0.17	0.79 ± 0.18	0.80 ± 0.18	0.82 ± 0.16
Parietal	0.72 ± 0.18	0.81 ± 0.14	0.77 ± 0.16	0.83 ± 0.13
Occipital	**0.71 ± 0.14** ^**a**^	0.85 ± 0.18	0.75 ± 0.15	0.86 ± 0.15
Anterior temporal	0.77 ± 0.19	0.77 ± 0.17	0.83 ± 0.17	0.81 ± 0.14
Middle temporal	0.77 ± 0.19	0.78 ± 0.21	0.82 ± 0.18	0.83 ± 0.13
Posterior temporal	0.72 ± 0.17	0.78 ± 0.20	0.78 ± 0.16	0.84 ± 0.13
LZC				
Prefrontal pole	**0.36 ± 0.05** ^**a**^	0.44 ± 0.94	**0.38 ± 0.06** ^**b**^	0.46 ± 0.11
Frontal	0.41 ± 0.11	0.50 ± 0.18	**0.44 ± 0.10** ^**b**^	0.53 ± 0.13
Center	**0.38 ± 0.09** ^**a**^	0.47 ± 0.13	0.41 ± 0.12	0.49 ± 0.11
Parietal	**0.38 ± 0.11** ^**a**^	0.50 ± 0.12	**0.41 ± 0.11** ^**b**^	0.52 ± 0.13
Occipital	**0.37 ± 0.10** ^**a**^	0.51 ± 0.14	**0.39 ± 0.11** ^**b**^	0.51 ± 0.15
Anterior temporal	0.40 ± 0.12	0.43 ± 0.13	0.44 ± 0.12	0.46 ± 0.13
Middle temporal	0.39 ± 0.10	0.47 ± 0.16	0.43 ± 0.12	0.49 ± 0.15
Posterior temporal	**0.38 ± 0.10** ^**a**^	0.48 ± 0.15	**0.40 ± 0.11** ^**b**^	0.49 ± 0.13
D2				
Prefrontal pole	3.31 ± 0.37	3.61 ± 0.53	3.47 ± 0.39	3.68 ± 0.51
Frontal	3.55 ± 0.50	3.67 ± 0.75	3.67 ± 0.52	3.83 ± 0.75
Center	3.44 ± 0.49	3.68 ± 0.64	3.66 ± 0.59	3.74 ± 0.62
Parietal	3.40 ± 0.54	3.77 ± 0.54	3.57 ± 0.58	3.73 ± 0.58
Occipital	**3.38 ± 0.50** ^**a**^	3.97 ± 0.73	3.53 ± 0.55	3.85 ± 0.72
Anterior temporal	3.51 ± 0.60	3.64 ± 0.74	3.70 ± 0.60	3.70 ± 0.57
Middle temporal	3.51 ± 0.61	3.78 ± 0.82	3.70 ± 0.64	3.91 ± 0.77
Posterior temporal	3.47 ± 0.57	3.75 ± 0.69	3.61 ± 0.60	3.68 ± 0.54

VS/UWS, Vegetative state/unresponsive wakefulness syndrome; MCS, Minimally conscious state; LE, Lyapunov Exponent; ApEn, Approximate Entropy; LZC, Lempel–Ziv Complexity; D2, Correlation Dimension D2. Statistical ratios: The ratio of MCS- to MCS+ in resting state, Bold ^a^*p* < 0.05. The ratio of MCS- to MCS+ in preferred music, Bold ^b^*p* < 0.05.

### 3.5 Correlation between the total CRS-R score and nonlinear algorithm metrics across brain regions

**Figure 2:**
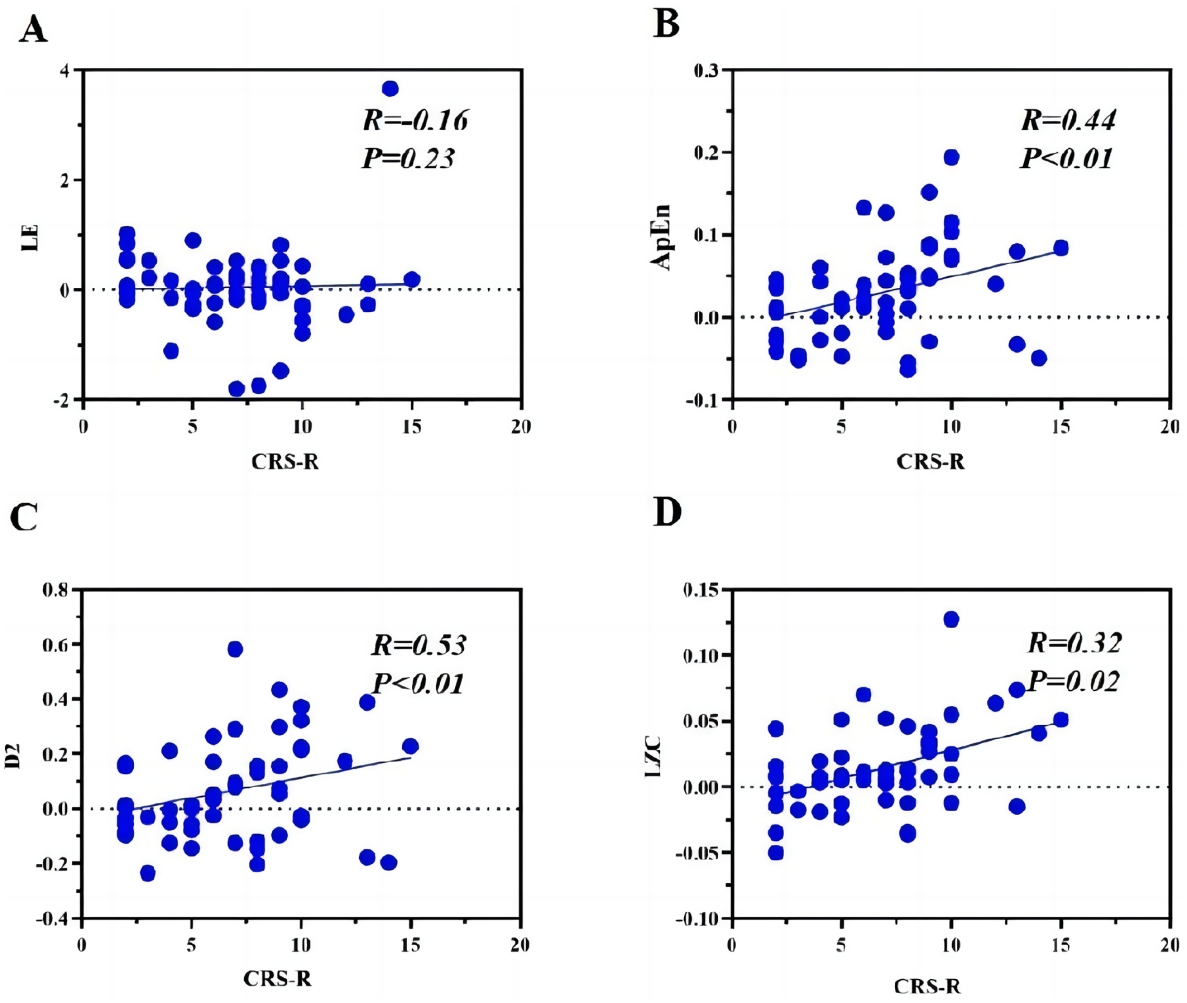
Correlation analysis. Scatter plots showing Pearson correlations between consciousness levels (CRS-R scores) and EEG complexity measures in DOC patients. Each panel displays: **(A)** Lyapunov exponent (LE: *R* = -0.16, *p* = 0.23); **(B)** Approximate entropy (ApEn: *R* = 0.44, *p* < 0.01); **(C)** Correlation dimension (D2: *R* = 0.53, *p* < 0.01); **(D)** Lempel-Ziv complexity (LZC: *R* = 0.32, *p* = 0.02). VS/UWS, Vegetative state/unresponsive wakefulness syndrome; MCS, Minimally conscious state.

This study employed correlation analysis to investigate the relationship between the total CRS-R scores of patients with DOC and the mean values of nonlinear algorithm metrics across different brain regions in response to musical stimulation. The differences in responses to musical stimuli were used to assess the patient’s ability to perceive external stimuli. The correlation between the differences in musical stimuli obtained using the LE algorithm and the total CRS-R score was not significant (*R* = −0.16, *p* = 0.23) (Figure 2A). In comparison, the other three metrics showed significant correlations with the total CRS-R score. The moderate correlation was observed for LZC (*R* = 0.53, *p* < 0.01; Figure 2C), followed by ApEn (*R* = 0.44, p < 0.01; Figure 2B), and the lowest significant correlation was found for D2 (*R* = 0.32, *p* = 0.02; Figure 2D).

## 4 Discussion

The brain, as a complex neural system, often exhibits chaotic firing behaviors in response to stimuli, which can be observed in EEG signals that possess non-smooth and non-linear characteristics (Liang et al., 2020; Altıntop et al., 2022). Traditional linear analysis methods, which treat EEG signals as smooth and linear, may overlook intrinsic information pertinent to DOC, such as the phase correlations between different brain regions and the hierarchical organization of neural networks. Nonlinear dynamical systems, characterized by “chaos,” are particularly suited for exploring biological time series, especially EEG (Akdemir Akar et al., 2015). Chaos theory and nonlinear dynamical systems theory address deterministic systems that exhibit complex and seemingly random behavior (Kargarnovin et al., 2023). Therefore, the present study examined the excitability of the cerebral cortex in patients with DOC in response to musical stimuli, using various nonlinear dynamical parameters. Three main results were obtained: 1. Preferred music stimuli induced excitability in the cerebral cortex of patients with DOC, and more brain regions were excited in the MCS group than in the VS/UWS group; 2. Different nonlinear kinetic algorithms produced different results; however, their overall trends were almost identical. LE, ApEn, LZC, and D2 were lower in both the VS/UWS and MCS groups than in the healthy control group, with the lowest values in the VS/UWS group, followed by the MCS group 3. In addition to LE, changes in ApEn, LZC, and D2 induced by preferred music stimuli in patients with DOC were significantly correlated with the CRS-R scores (*p* < 0.05).

Patients with DOC often have visual impairments, however their auditory systems are typically intact (Magee et al., 2017; Yu et al., 2025). Therefore, in the present study, preferred music was used to stimulate responses to external stimuli in patients with DOC. Musical stimulation is expected to increase activity in the frontal, temporal, parietal, and subcortical areas, potentially positively affecting recovery of consciousness (Li et al., 2020). The study’s findings, using nonlinear analysis with ApEn, LZC, and D2 algorithms, consistently revealed that musical stimulation activated the prefrontal, frontal, central, and temporal lobe regions in the MCS group. In contrast, no significant activation was observed in any brain region in the VS/UWS group. Healthy participants exhibited significant activation in the prefrontal pole, frontal, parietal, occipital, and temporal regions, as revealed by the ApEn algorithm when exposed to their most preferred music. In addition, the MCS group demonstrated higher responsiveness to music than did the VS/UWS group, correlating with the CRS-R total score. The overall trend showed lower values in both the VS/UWS and MCS groups than in healthy participants, with the MCS- group exhibiting lower values than the MCS + group in the central, parietal, and occipital lobes. These results are consistent with the findings of Liu et al. (2021), Zhang et al. (2021), Liu et al. (2022) and other experts, such as Ling et al. (2023), suggesting that the reduction in nonlinear indicators representing complexity (LE, ApEn, LZC, and D2) reflects a decrease in degrees of freedom. This reduction indicates a decline in coupled EEG rhythms and the dynamic response of the brain to external stimuli (Wu et al., 2011).

In contrast, the LE algorithm showed no statistically significant differences among all brain regions in the MCS group after musical stimulation, whereas patients in the VS/UWS group exhibited a significant increase in frontal and mesial temporal cortex complexities (*p* < 0.05). Correlation analysis also indicated an absence of significant relationship between the LE changes induced by music stimulation and the total CRS-R score in patients with DOC (*p* > 0.05). This differential sensitivity of LE between patient groups may reflect inherent limitations in nonlinear analysis: while LE can characterize low-dimensional deterministic dynamics, its reliability likely decreases for high-dimensional stochastic processes like EEG signals originating from large-scale neuronal networks (approximately 10⁵–10^8^) (Ma et al., 2021; Hart, 2024). As suggested by Pritchard (Pritchard et al., 1995) and other scholars, EEG signals are chaotic high-dimensional nonlinear systems. In patients with MCS, preserved thalamocortical connectivity might maintain high-dimensional EEG activity that exceeds LE’s detection capacity during complex musical processing, potentially accounting for the lack of significant LE changes. Conversely, in patients with VS/UWS—particularly those with HIE—may demonstrate reduced-dimensional dynamics due to thalamocortical disruption, potentially allowing LE to track residual neural oscillations more effectively. Based on these observations, we hypothesize that VS/UWS patients might have lower dimensionality of EEG signals compared to MCS patients in association with their poor state of consciousness, suggesting that LE suggesting could be more sensitive to signal changes in VS/UWS patients during music assessment. These interpretations, while theoretically framed, remain speculative and warrant further validation to establish their clinical relevance.

In recent years, ApEn and LZC values have been widely used by scholars worldwide to assess the state of consciousness of patients with DOC (Zhang et al., 2021; Qu et al., 2024). The results of this study suggest that both ApEn and LZC nonlinear analysis methods accurately reveal the specific cortical excitatory effects of musical stimulation on patients in the VS/UWS and MCS groups and that the MCS group showed a better ability to process music than the VS/UWS group. In addition, the differences in ApEn and LZC responses to musical stimulation significantly correlated with the total CRS-R scores, with LZC showing a slightly higher correlation than ApEn. Therefore, the present study concluded that both ApEn and LZC are valuable tools for applying nonlinear dynamics to explore brain function. Liu et al. (2023) found that permutation LZC had a better ability to distinguish between the VS/UWS and MCS states than the ApEn values. This may be because ApEn reflects complexity through changes in the EEG signal amplitude, which relies on the data length and amplitude information, making it more susceptible to noise artifacts. LZC, the other hand, reveals the degree of randomness in the EEG signal sequence. Unlike ApEn, which measures the unpredictability of information content in a time series, LZC determines the minimum information required to reconstruct the original signal. It is computationally fast and resistant to interference (Liang et al., 2020). This algorithmic difference explains the higher correlation between the LZC and total CRS-R scores compared with ApEn. However, unlike the results of the present study, Wu et al. (2011) found that music stimulation did not significantly elicit excitability in the cerebral cortex of patients with VS/UWS and MCS (*p* > 0.05) using ApEn and LZC nonlinear analysis methods. This discrepancy may be due to differences in the patient groups or the type of music selected. Compared with the popular music selected by Wu et al., the selection of preferred music in this study may have properties such as self-referentiality and connections to past emotional experiences, which are more capable of evoking a wide range of responses in the cerebral cortex of patients (Heine et al., 2015; Heine et al., 2017).

D2 is often interpreted as a measure of the complexity (or flexibility) of information processing related to alertness and mental activation levels and is a sensitive parameter in the nonlinear analysis of EEG (Carlino et al., 2012). In this study, D2 was used for the first time to assess the DOC. The difference in D2 evoked by auditory stimuli correlated well with the CRS-R scores (*R* = 0.32, *p* = 0.02). D2 revealed the effect of musical stimulation on brain area complexity, which was similar to the results observed for ApEn and LZC. Kargarnovin et al. (2023) found that the nonlinear kinetic parameters of healthy participants’ EEG were lower during musical stimulation (rock and light music) than during the resting state. This contradicts the results of the present study, in which healthy participants showed increased levels of ApEn, LE, and D2 upon stimulation with their preferred music. We hypothesize that this discrepancy could be due to the type of music involving participants’ past emotional experiences, which may better stimulate cortical excitability. Additionally, the inclusion of a heterogeneous group of participants (with different occupations and cultural backgrounds) could result in varied sensitivities to music, leading to differing outcomes. In the present study, the median D2 mean value of each brain region in the quiet state was 3 for both VS/UWS and MCS patients, which was lower than the D2 values of the healthy participants in the quiet state observed in the above study. The reduced consciousness in patients with DOC manifests as slower and more regularized cortical alertness and information processing, possibly accounting for their lower D2 values. Molnár et al. (1997) explored a patient with stroke using point-correlation dimension and found a low-dimensional region in the parietal region of his loss area, consistent with a relative decrease in EEG activity in the fast *γ*-band of CT scans, indicating that a nonlinear approach based on chaos theory can improve the sensitivity of electrophysiological methods for detecting cortical dysfunction.

Notably, language-based assessment and treatment are often insufficient to restore consciousness and cognitive abilities in patients with DOC because of the prevalence of language deficits in this population (Hu et al., 2021). Music, among various auditory stimuli, has unique self-referential properties, emotional valence, and affective qualities that can bypass language barriers (Li et al., 2020). Therefore, music therapy has been used as an initial treatment to help patients with DOC regain consciousness (Park and Davis, 2016; Li et al., 2020). The present study assessed the cerebral cortex response to musical stimuli in VS/UWS, MCS (MCS- and MCS+), and healthy participants using four nonlinear metrics. Although the results varied, the overall trend indicated a higher cortical complexity after auditory stimulation in healthy participants than in patients with MCS and VS/UWS. This suggests that using preferred musical stimuli as an experimental paradigm has the potential to assess the state of consciousness in diagnosing patients with DOC. Additionally, the study’s finding that music stimulation increases cortical excitability in patients with DOC implies that those who exhibit nonlinear kinetic changes due to music stimulation should receive active therapeutic interventions, including pharmacological and neurophysiological treatments (Hu et al., 2021; Wu et al., 2024).

Four key limitations of this study merit discussion. First, while CRS-R behavioral assessment remains the diagnostic standard for DOC, motor/language impairments and consciousness fluctuations contribute to significant misdiagnosis risks. Although our protocol included triple assessments performed by an experienced neurophysiologist, this approach may still miss covert consciousness in patients with MCS. Future studies should implement repeated dual-examiner evaluations to improve diagnostic accuracy. Second, the subgroup analysis was limited by small and unbalanced sample sizes (MCS-: *n* = 17 vs. MCS+: *n* = 12), particularly for the clinically rare MCS + group. Despite including all available cases, statistical power may be inadequate. Multicenter studies are needed for validation. Third, the use of patient-preferred music enhances stimulus personalization through autobiographical relevance, yet presents methodological limitations: (1) predominant use of popular music (>85%) limits genre-specific analysis; (2) uncontrolled musical parameters and lack of no-music controls constrain mechanistic interpretations. Future studies should: (i) compare music genres systematically, and (ii) implement controlled paradigms with psychoacoustically-matched stimuli and multi-condition baselines (silent/non-musical/neutral-music) to isolate music-specific neural responses. Fourth, the age range of participants was broad (18–80 years old). Due to the small sample size, we could not perform age stratification as suggested by Ezaki et al. (2018) (18–30 years old, 30–60 years old, 60–80 years old). Future studies should expand sample sizes to examine age-specific effects (18–30, 30–60, 60–80 years) on EEG nonlinear parameters across quiet and music conditions in DOC patients versus controls.

## 5 Conclusion

This study, utilizing nonlinear kinetic parameters of chaos theory, demonstrated that preferred music could increase cerebral cortex complexity in patients with DOC. This finding provides clinicians with valuable insights for selecting auditory stimulation modalities. The nonlinear parameters (ApEn, LZC, and D2) showed significant correlations with CRS-R scores and may serve as auxiliary indicators for consciousness assessment, while LE exhibited potential in capturing low-dimensional signal changes in VS/UWS patients during musical stimulation. These results suggest that combining these four parameters could offer a more comprehensive evaluation framework for DOC states. Future studies should: (1) employ multimodal neurophysiological tools to validate these nonlinear measures, and (2) establish their clinical utility through longitudinal intervention studies. While current findings demonstrate correlational relationships between nonlinear parameters and clinical assessments, the interpretation of specific neural mechanisms requires caution. In conclusion, the application of EEG nonlinear kinetic parameters in DOC is still in its exploratory phase. While some information remains challenging to interpret, nonlinear kinetic analysis technology holds promise for enhancing clinical practices by identifying effective interventions and providing a deeper understanding of brain function in patients with DOC.

## Data Availability

The raw data supporting the conclusions of this article will be made available by the authors, without undue reservation.
